# Gastric mucormycosis complicated by a gastropleural fistula: A case report and review of the literature

**DOI:** 10.1097/MD.0000000000018142

**Published:** 2019-11-27

**Authors:** Tomohisa Uchida, Momoko Okamoto, Keita Fujikawa, Daisuke Yoshikawa, Akinari Mizokami, Tomo Mihara, Akira Kondo, Kazuo Ohba, Kazuhiro Kurohama, Masahiro Nakashima, Ichiro Sekine, Shigeki Nakamura, Yoshitsugu Miyazaki, Atsushi Kawakami

**Affiliations:** a Department of Rheumatology; b Department of Gastroenterology; c Department of Infectious Disease, Japan Community Healthcare Organization, Isahaya General Hospital, Isahaya; d Department of Tumor and Diagnostic Pathology, Atomic Bomb Disease Institute, Nagasaki University, Nagasaki; e Department of Chemotherapy and Mycoses, National Institute of Infectious Diseases, Tokyo; f Department of Immunology and Rheumatology, Graduate School of Biomedical Sciences, Nagasaki University, Nagasaki, Japan.

**Keywords:** adult-onset Still disease, gastric mucormycosis, gastropleural fistula, tocilizumab

## Abstract

**Rationale:**

Mucormycosis is a rare opportunistic fungal infection with poor prognosis. The incidence of mucormycosis has been increasing, and it is a threat to immunocompromised hosts. We present a case of gastric mucormycosis complicated by a gastropleural fistula during immunosuppressive treatment for adult-onset Still disease (AOSD).

**Patient concerns:**

An 82-year-old woman diagnosed with AOSD who developed gastric ulcers during the administration of an immunosuppressive therapy with corticosteroids, cyclosporine, and tocilizumab complained of melena and epigastralgia. Esophagogastroduodenoscopy showed multiple ulcers covered with grayish or greenish exudates.

**Diagnoses:**

The patient diagnosed with mucormycosis based on culture and biopsy of the ulcers, which showed nonseptate hyphae branching at wide angles. *Mucor indicus* was identified using polymerase chain reaction.

**Interventions and outcomes:**

Although liposomal amphotericin B was administered, gastric mucormycosis was found to be complicated by a gastropleural fistula. The patient died because of pneumonia due to cytomegalovirus infection, and autopsy revealed the presence of Mucorales around the fistula connecting the stomach and diaphragm.

**Lessons:**

Gastric mucormycosis is refractory to treatment and fatal. Surgical resection, if possible, along with antifungal drugs can result in better outcomes.

## 1 Introduction

Mucormycosis is a rare but fatal fungal infection caused by Mucorales with an acute course. Recently, as the incidence of mucormycosis has been increasing, it has been considered an important infectious disease observed among immunocompromised patients.^[1,2]^ Its common infection sites include the skin, paranasal sinus, and lungs, but gastrointestinal manifestations are rare.^[3]^ Mucormycosis is diagnosed based on histologic findings or positive culture from affected lesions owing to the lack of validated serologic biomarkers. Management guidelines for mucormycosis recommend a combination treatment with antifungal drugs and surgical resection of the devitalized tissue.^[4]^ Herein, we describe a case of gastric mucormycosis complicated by a gastropleural fistula during immunosuppressive treatment for adult-onset Still disease (AOSD).

## 2 Case report

**Figure 1:**
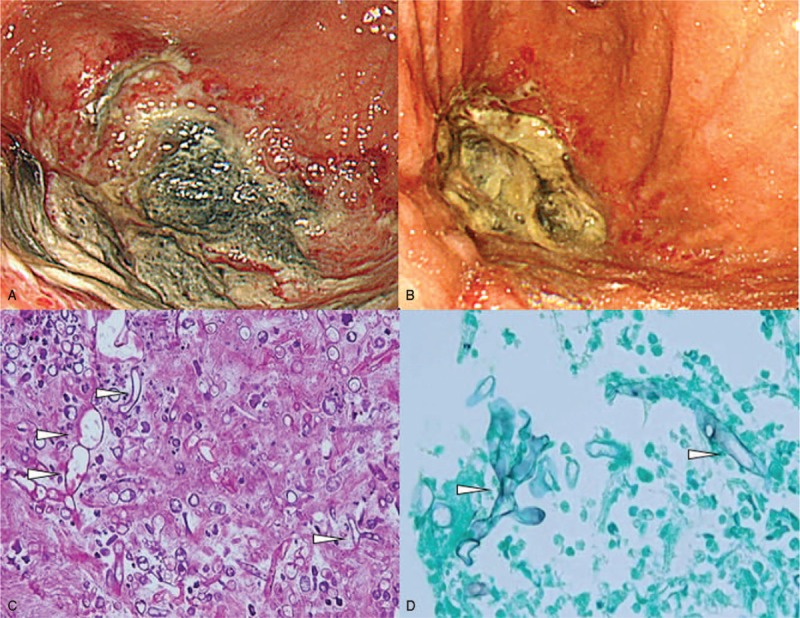
Esophagogastroduodenoscopy findings and biopsy specimens of the gastric mucosa. Necrotic ulcer is covered with gray-greenish exudate at the fornix of stomach (A). The size of the ulcer has reduced after administration of an antifungal drug (B). Histopathologic findings of the gastric ulcer following staining with hematoxylin and eosin stain (C) and Grocott methenamine silver stain (D). White arrowheads indicate nonseptate, right-angle, branched fungal hyphae.

**Figure 2:**
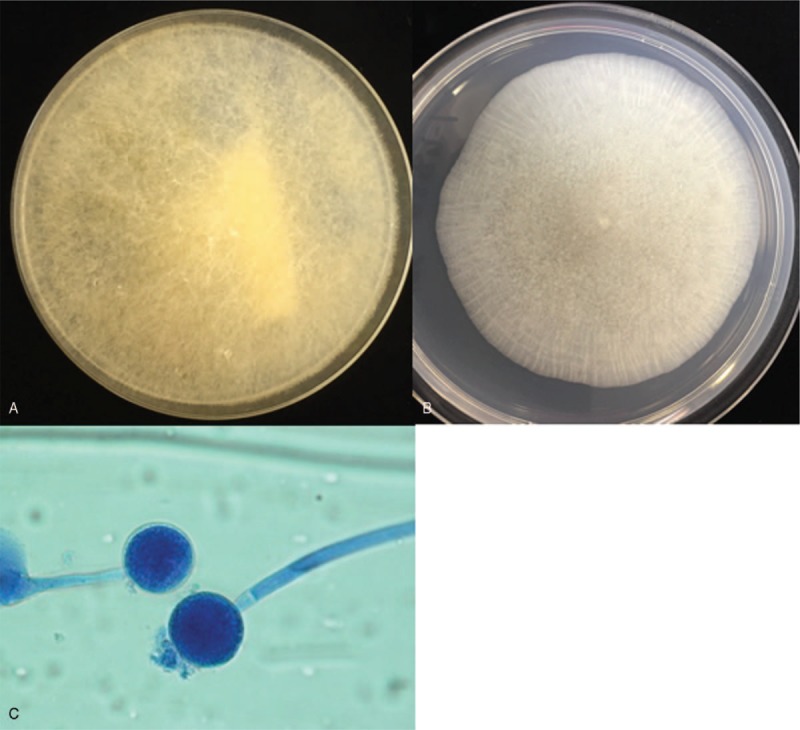
Cultures from exudates of gastric ulcers. Colony on Sabouraud dextrose agar plate cultured 35°C for 2 days and after that culturing at 25°C for 9 days (A). Colony on potato dextrose agar plate cultured at 30°C for 4 days (B). Direct mount from a culture fixed with lactophenol cotton blue at 1000× magnification (C).

**Figure 3:**
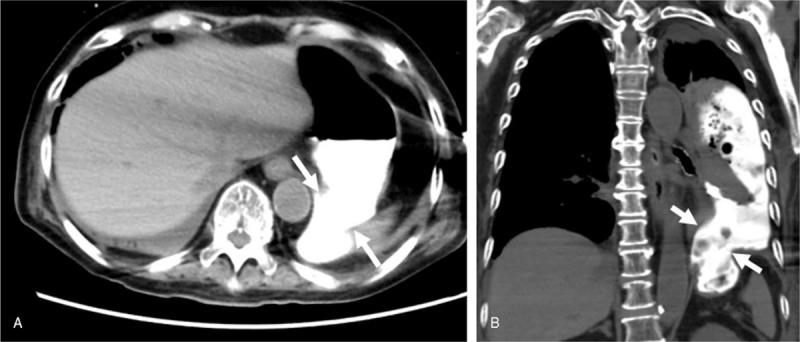
Chest computed tomography after administration of diluted amidotrizoate in the coronal (A) and transverse (B) planes. White arrows indicate the gastropleural fistula.

**Figure 4:**
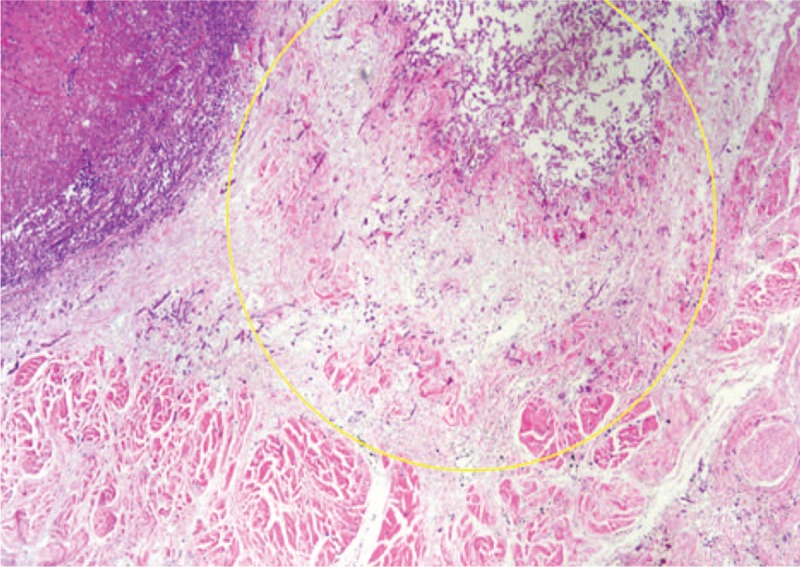
Histopathologic findings on autopsy. Yellow circle indicates invasions of Mucorales at the muscularis propria near the gastropleural fistula.

An 82-year-old woman developed fever, sore throat, general malaise, and polyarthralgia and was admitted to our hospital. At hospitalization, her vital signs were as follows: blood pressure, 109/84 mm Hg; pulse rate, 92 beats/min; and temperature, 37.3°C. She presented with cervical lymphadenopathy, salmon-colored rash on the torso and extremities, and bilateral tenderness of the shoulder, elbow, and ankle joints. She had no relevant medical and family histories. Laboratory data revealed a white blood cell count of 17,110/μL with 92% neutrophils, C-reactive protein (CRP) level of 28.57 mg/dL, serum ferritin level of 9899 ng/mL, and elevated liver enzyme levels. Rheumatoid factor, anticitrullinated protein antibody, and antinucleolar antibody were tested to be negative, and whole body computed tomography (CT) revealed no abnormalities. Subsequently, she was diagnosed with AOSD based on Yamaguchi criteria^[5]^ and was prescribed oral prednisolone (50 mg/d) and oral cyclosporine (200 mg/d). Her symptoms and serum ferritin levels remarkably improved on day 10. On day 36, she developed high fever with elevated CRP and serum ferritin levels. Because AOSD relapse was suspected, she was intravenously administered 400 mg tocilizumab (8 mg/kg) after methylprednisolone pulse therapy at a dose of 1 g/d for 3 days. This regimen improved her symptoms and serum ferritin levels. However, during this therapy, she complained of epigastralgia and melena, and esophagogastroduodenoscopy (EGD) showed peptic ulcers at the pylorus. Therefore, she was prescribed vonoprazan instead of lansoprazole, resulting in immediate symptomatic relief. However, epigastralgia and melena relapsed on day 47. On day 51, a repeat EGD showed multiple ulcers covered with grayish or greenish exudates spreading from the upper body of the stomach toward the fundus (Fig. 1A). Biopsy showed nonseptate hyphae branching at wide angles, indicative of the presence of Mucorales in the gastric mucosa (Fig. 1C) that were stained with Grocott methenamine silver (Fig. 1D). Colony obtained from exudates around gastric ulcers culturing on the Sabouraud dextrose agar at 35°C for 2 days and after that culturing at 25°C for 9 days was white and fuzzy mold colony (Fig. 2A). Colony on the potato dextrose agar plate incubated at 30°C for 4 days was fluffy and white (Fig. 2B). Nonseptate hyphae with round sporangia were observed microscopically (Fig. 2C). The internal transcribed spacer (ITS) region and D1/D2 region of the ribosomal RNA gene of the isolates were sequenced to provide further support.^[6,7]^ As a result, sequences of both the ITS and D1/D2 regions were found to have 100% similarity with *Mucor indicus*, CBS423.71. The patient was diagnosed with gastric mucormycosis, and intravenous liposomal amphotericin B (L-AMB) was administered at a dose of 200 mg/d (5 mg/kg/d). During the antifungal therapy, oral prednisolone was carefully tapered to 17.5 mg/d, but high serum ferritin levels were sustained between 1789 and 4010 ng/mL. On day 92, EGD revealed shrinking of the ulcers (Fig. 1B). However, the patient complained of left-sided chest pain on day 102, and chest CT identified a pneumothorax that was relieved by inserting a drainage tube into the chest cavity. Additionally, chest CT after the administration of diluted amidotrizoate showed it to be leaking from the stomach into the thoracic cavity, suggesting the presence of a gastropleural fistula (Fig. 3A, B). Surgical correction of the fistula was impossible considering the patient's worsening general condition. Although her thoracic cavity was drained and she was treated with broad-spectrum antibacterial drugs and L-AMB, she died pneumonia on day 159. Autopsy revealed Mucorales infection near the gastric perforation (Fig. 4), and cytomegalovirus pneumonia was considered the possible direct cause of death.

## 3 Discussion

We describe a case of gastric mucormycosis secondary to immunosuppressive treatment for AOSD. Although the patient was treated with antifungal drugs, the gastric ulcer developed into a gastropleural fistula and the patient died due to pneumonia. Autopsy identified the cause of the fistula to be gastric perforation due to the spread of Mucorales infection from gastric ulcers.

Mucormycosis is an acute and fatal fungal infection with a mortality rate of 54% in the world.^[3]^ The risk factors for mucormycosis primarily include neutropenia, diabetes mellitus (DM, especially with ketoacidosis), malignancies, hematopoietic stem-cell transplantation, solitary organ transplantation (SOT), corticosteroid use, iron overload, deferoxamine use, and trauma.^[8]^ In addition, there are several case reports on mucormycosis in patients undergoing biologic therapies,^[9,10]^ for example, one report describes the presence of pulmonary mucormycosis during treatment with tocilizumab.^[11]^ In the case described here, the immunosuppressive therapy administered with high-dose corticosteroids, cyclosporine A, and tocilizumab is a possible risk factor for mucormycosis.

Gastrointestinal manifestations of mucormycosis account for only 7% of all cases,^[3]^ with the stomach being the most frequently affected organ.^[8]^ We reviewed 20 reports on gastric mucormycosis (Table 1).^[12–30]^ We identified common symptoms to be melena, hematemesis, and abdominal distention or pain, and most patients had predisposing factors such as DM, SOT, or corticosteroid use. The common site of infection is the upper body of the stomach, and the ulcers have been characterized as having greenish, grayish, or black exudate or necrosis during EGD.

**Table 1:**
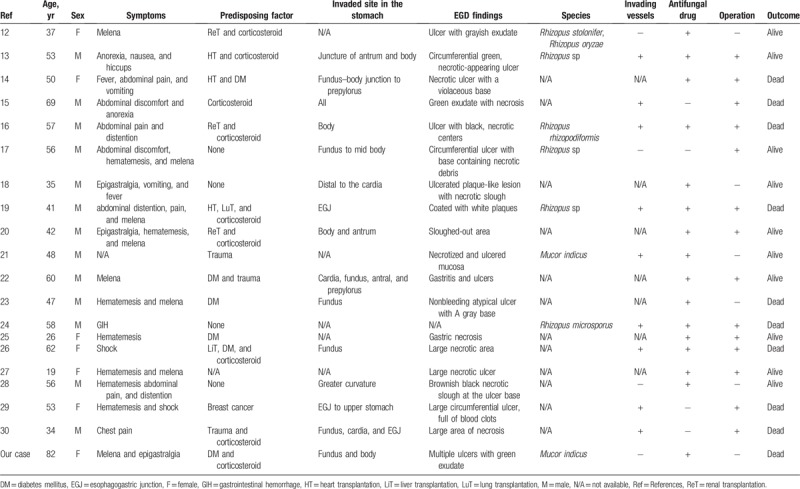
A review of previous reports and our case regarding gastric mucormycosis.

Angioinvasion is a characteristic of mucormycosis progression, which probably contributes to its hematogenous dissemination to other organs.^[31]^ Our literature review revealed that 7 of the 9 cases with vessel invasions as per histologic examination resulted in death, suggesting that vessel invasion is a poor prognostic factor. Almyroudis et al reported that a combination of surgery with AMB therapy yielded a better outcome than AMB administration alone in SOT recipients, with mortality rates being 34.3% and 62.5%, respectively.^[32]^ However, during literature review, we found that 5 of 10 cases treated with antifungal drugs and surgical intervention resulted in death. Thus, it is possible that gastric mucormycosis has poor prognosis even with the combined use of surgical treatment and antifungal drug therapy. Notably, we found only 1 case report that described a gastropleural fistula secondary to gastric mucormycosis.^[30]^ In this report, an HIV-positive male developed gastric mucormycosis after experiencing an abdominal trauma, and a postoperative gastropleural fistula developed due to gastric perforation. In our case, the ulcers initially seemed to have improved upon treatment with an antifungal drug, but the outcome remained unfavorable. Nonetheless, it is possible that a combination of surgical resection for gastric mucormycosis and antifungal therapy during the early stages of the disease improves outcomes and prognosis.

Mucorales require host iron for hyphal growth during host cell invasion,^[33]^ and 10 of the 20 reported cases on gastric mucormycosis involved peptic ulcers.^[34]^ Possibly, gastric bleeding from peptic ulcers provides iron to Mucorales for growth and a route for angioinvasion. Additionally, hyperferritinemia is a reported poor prognostic factor for mucormycosis,^[35]^ and inflammation due to infection increases storage iron levels through various cytokines and hepcidin.^[36]^ Thus, hyperferritinemia may reflect inflammation due to mucormycosis. Meanwhile, hyperferritinemia mirrors the disease activity of AOSD and is a risk factor for macrophage activation syndrome.^[37]^ In our case, hyperferritinemia was sustained during the clinical course, and it was difficult to distinguish between the disease activity of AOSD and inflammation due to mucormycosis. In such cases, we are of the opinion that the significance of serum ferritin levels should be carefully judged based on clinical course.

In conclusion, we reported a case of gastric mucormycosis complicated by a gastropleural fistula. In an immunocompromised host, gastric mucormycosis should be considered a cause of refractory gastric ulcers, and it is recommended to confirm Mucorales infection by gastric biopsy and culture of exudates. A mucormycosis is a fatal fungal infection, and antifungal drugs and timely surgical resection are important to manage the disease.

## Acknowledgment

The authors thank the patient and the family as well as the medical staff for their contribution to the case report.

## Author contributions

**Investigation:** Momoko Okamoto, Daisuke Yoshikawa, Tomo Mihara, Akira Kondo, Kazuhiro Kurohama, Masahiro Nakashima, Ichiro Sekine, Shigeki Nakamura.

**Supervision:** Daisuke Yoshikawa, Akinari Mizokami, Kazuo Ohba, Yoshitsugu Miyazaki, Atsushi Kawakami.

**Writing – original draft:** Tomohisa Uchida.

**Writing – review & editing:** Keita Fujikawa.

Keita Fujikawa orcid: 0000-0003-2907-5359.
